# myEpi. Epidemiology of One

**DOI:** 10.3389/fpubh.2014.00097

**Published:** 2014-08-12

**Authors:** Georgiy Bobashev

**Affiliations:** ^1^RTI International, Durham, NC, USA

**Keywords:** mobile health, epidemiology, statistics, data science, myEpi, wearable devices, evidence-based practice, self-care

## Abstract

A new concept of within-individual epidemiology termed “myEpi” is introduced. It is argued that traditional epidemiological methods, which are usually applied to populations of humans, can be applicable to a single individual and thus used for self-monitoring and forecasting of “epidemic” outbreaks within an individual. Traditional epidemiology requires that results be generalizable to a predefined population. The key component of myEpi is that a single individual may be viewed as an entire population of events and thus, the analysis should be generalizable to this population. Applications of myEpi are aimed for, but not limited to, the analysis of data collected by individuals with the help of wearable sensors and digital diaries. These data can include physiological measures and records of healthy and risky behaviors (e.g., exercise, sleep, smoking, food consumption, alcohol, and drug use). Although many examples of within-individual epidemiology exist, there is a pressing need for systematic guidance to the analysis and interpretation of intensive individual-level data. myEpi serves this need by adapting statistical methods (e.g., regressions, hierarchical models, survival analysis, agent-based models) to individual-level data.

## Epidemiology and myEpi

*Epidemiology*, literally “the study of what is upon the people,” is defined as the science of the incidence, distribution, and control of disease conditions in a population (1). Epidemiology provides public health and policy makers with evidence such as risk factors for disease and targets for preventive medicine. Epidemiology does not focus on a specific individual; treating disease conditions of an individual is traditionally left to the clinical field. At the same time, more individuals are showing active interest in their own health; and more physicians are interested in incorporating the wealth of existing information into their practices. When the focus is on the health and behavior of a specific individual, use of epidemiological methods can lead to confusion. On one hand, evidence-based principles state that practical decisions should be based on research studies. In fact, knowing that smoking leads to a plethora of health conditions including cancer is a good motivation for an individual to quit smoking, and for a never-smoker not to initiate. On the other hand, overreliance on population-level evidence can lead to a well-known ecological fallacy when health risks (or lack thereof) are erroneously projected onto an individual. For example, lawsuits have been initiated when a physician relies on evidence and assumes low risk and a patient develops a health condition (2).

**Figure 1:**
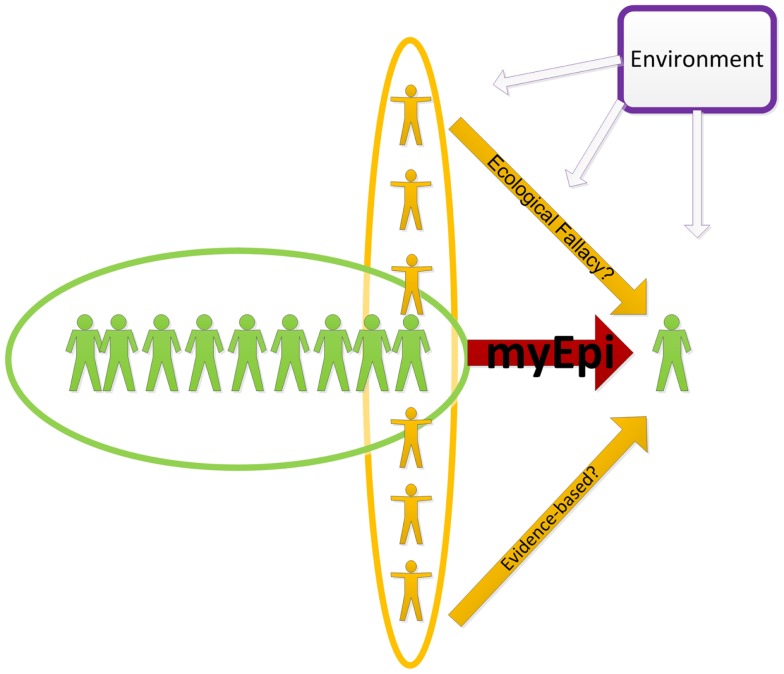
**An illustration of inferring to an individual form the population-level and individual-level data**. The green “myEpi” oval denotes within-person data which could be also combined with information about blood relatives, social networks, as well as the entire population and the environment. The purpose of the analysis, however, is the individual-level inference.

In this article, I introduce the concept of individual epidemiology (myEpi), which aims to provide individual-level inference. By definition, myEpi considers an individual as a population of person-level events and behaviors (Figure 1) and thus inferences about an individual can be obtained from within-person data.

MyEpi does not ignore evidence collected from others in the population, in fact it embraces these data in the same way epidemiological studies embrace environmental factors. In this sense, the polluted environment of a faulty chemical plant could contribute to the increased risk of disease in a neighboring town, similar to the way disease status of close relatives (genetics) or social networks (behavioral influence) contributes to an increase in disease risk for an individual.

## Available Data

Over the last decade, a revolution in data collection within an individual (e.g., electronic diaries, ecological momentary assessments, and multiple physiological sensors) has allowed for the collection of large amounts of “intensive” data (3). Although traditional methods such as Timeline Followback (4) are still being used for research purposes, web, and mobile applications allow one to record and analyze data in real time. Although the design, quality, and ownership of the data remain subjects of evolving research and policies, one thing is evident: data collected by an individual and for the use by that individual can have a strong impact on that person’s health and behavior.

Statistical requirements for myEpi data follow the same statistical power rules as a traditional epidemiological study; a longitudinal study with sufficient observations could potentially be used for helping a specific patient. In decision sciences, the requirements for the amount of data can be relaxed in exceptional cases when individual data carry critical information. For example, in a hypothetical situation three antibiotics (A, B, and C) are available to treat a patient. If a patient developed severe allergic reaction in the past to drugs A and B on two separate occasions, then the only available option is C. This decision is likely to be made regardless of whether population-level clinical trials show that drug B is most efficacious. In this example, the presence of only two observations is sufficient to rule out two of three antibiotics.

This example also illustrates that data for medical decision purposes should be of high quality. In reality, without the availability of electronic health records, healthcare providers often rely on patients’ recollections of past events, often with a long time lag and thus reduced reliability. Additionally, medical records often do not include events that happen outside health care. In addition to and sometimes instead of formal healthcare individuals sometimes rely on personal opinion and experience and the advice of family, influential social network members, advertisement, etc. (5, 6). Recordings of self-treatment and outcomes become more available and should be used to provide insights into individuals’ health and behavior.

## Available Analysis Methods

These within-person data are different from data traditionally collected in epidemiological studies, but myEpi can adapt most methodology from traditional epidemiological methods. Below are a few examples. When collecting multiple observations per subject, epidemiologists have studied between- and within-subject variation for decades and used regression-based tools like Hierarchical Linear Models. In epidemiological studies within-subject variation represents the average over many individuals rather than the variation within a single individual. In myEpi, the focus is on variability within an individual (3, 7–9). Bayesian methods open an opportunity to combine evidence from population-level research with observed data from a specific individual, thus borrowing strength from the group where actual data are lacking (10).

**Figure 2:**
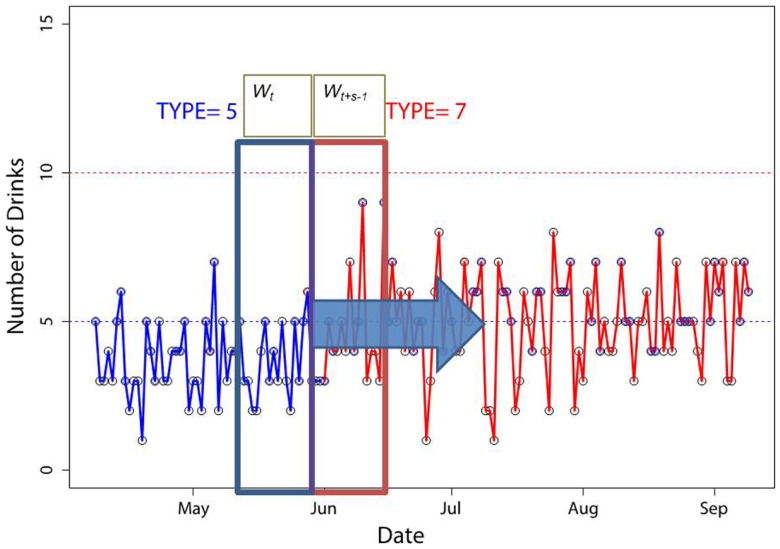
**A “moving windows” method to identify patterns of alcohol use trajectories**. Distributional properties of sliding windows *W*_t_ and *W_t_*_+_*_s_*_−1_ are compared to each other. The point when the distributions become significantly different signifies the change in patterns. We illustrate the point at which the pattern switched from type 5 to 7 as the number of drinks increases. The figure is reproduced with permission from Ref. (8).

Infectious diseases such as measles and influenza have been extensively studied using epidemic surveillance and mathematical models (11). These models are based on daily or regular incidence reports and if model parameters are well calibrated the model can predict the course of an epidemic (11–13) and evaluate strategies to contain it (12, 13). The same methods, such as distinguishing an emerging epidemic from occasional outbreaks, can be used when considering individual data. The application again requires a change of mindset. Similar to considering regular (e.g., weekly or monthly) disease incidence reports one can consider regular (e.g., daily or weekly) reports of specific events. These events could be categorical (e.g., exercised or not), count (e.g., drank five beers), or continuous (e.g., consumed 3680 calories). In substance use studies, a number of tools are available to track alcohol and tobacco consumption; drawing an analogy with an infectious epidemic, the tools can detect the start of increased use (8) (Figure 2), predict most likely moment for relapse (14), predict future use (15), and identify strategies to influence the recovery process (16, 17).

**Figure 3:**
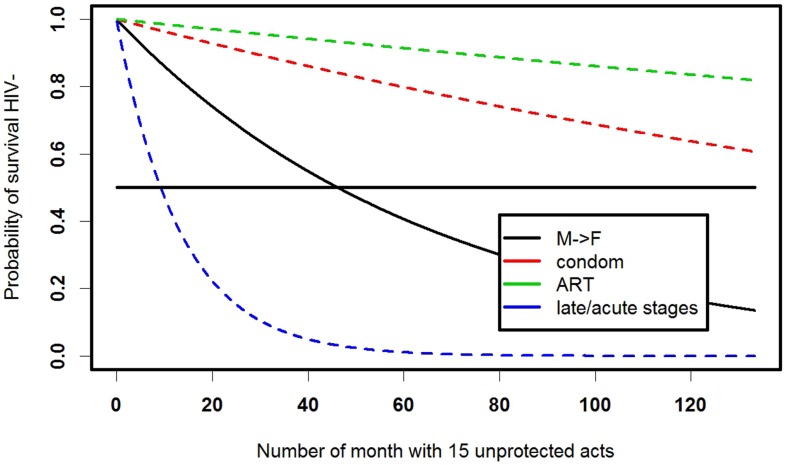
**Survival curves for staying HIV negative for a female with a male HIV-positive partner in a latent HIV stage, assuming two unprotected vaginal intercourses per week**. Model details and parameters are described in detail in Ref. (7).

Survival analysis has been broadly used to estimate exposure risks in the population and survival curves can show, for example, survival from HIV among men who have sex with men in a long-term cohort study (18). Although the study emphasized that condoms and antiretroviral treatment (ART) work as preventive factors, it tells little about individuals’ chances to seroconvert. At the same time, knowledge of individual sexual and drug-using behavior in discordant couples allows one to estimate the timing of HIV seroconversion (7). Changes in individual behavior (e.g., compliance with ART) alter the HIV-negative survival of a partner (Figure 3).

For complex problems involving health systems and adaptive behavior computational methods become very popular (9, 19). Agent-based models (ABMs) have recently become powerful tools to estimate the effects of interventions on community and population health (20, 21). At the same time, ABMs allow one to track an individual and examine what could potentially happen to this specific individual over the course of time under different scenarios (22). Modeling an individual in the context of social network and a series of individual events is in its infancy, especially when considering health outcomes. However, this area has been progressively developed in the context of military actions and response to terrorism (23).

## Interpretation of Data Summaries and Behavior Change

Besides professionally designed and analyzed studies, individuals collect large amounts of data for their personal interpretation and decision making. Machine learning tools that are being developed as part of the “Big Data” research agenda can provide people with real-time interpretations of their data. Incorporation of these tools in the backbone of mobile apps is likely to play a crucial role in the future. At the same time, understanding probability, associations, causality, and uncertainty to the users of thousands of web and mobile applications becomes critical because these interpretations affect health and behavior decisions. Just as a public health policymaker needs to understand and interpret epidemiology, an individual needs to understand and interpret individual data for personal decisions.

An individual can also compare him- or her-self to other individuals, trends, and percentiles in the available sample. In this sense, such analysis is similar to how cross-cultural and cross-country comparisons are made in epidemiology. However, myEpi emphasizes considering these trends and percentiles only as a context for an individual trend. For example, if an individual is monitoring his or her blood pressure he or she might also like to compare it with national statistics, daily and annual trends, etc. However, the most important comparison is with norms for self. Some people can naturally have lower blood pressure and a sudden jump in pressure can be worrisome even if it remains below the national norm. Analogously, an infant can develop perfectly well according to her own growth curve, which might be in a low or high national percentile.

## Control of Personal Health

Control of national epidemics has been a prerogative of the government’s public health system. Control of individual health, however, has been more ambiguous, with a large role placed on physicians and health care providers in general but mostly for the treatment of disease. Prevention has traditionally been a part of individuals’ everyday life. At the same time, public health officials and physicians have been trained for years to recognize patterns within populations, groups of patients, and a single individual, while the majority of individuals are not trained to monitor themselves in a systematic way. This lack of statistical training often results in superstitions and ungrounded beliefs. Currently, the availability of data collecting and monitoring tools could potentially lead to more ungrounded beliefs and erroneous conclusions if rigorous and reliable help is not provided to individuals for analysis of their own data.

## Link between Epidemiology and myEpi

One of the disputed issues in the use of individual data is their generalizability to a larger population. Under the myEpi concept, models and methods should not necessarily be generalizable to the population (it would be an additional merit if they were) but should show validity *within* an individual. This distinction becomes increasingly important in the process of funding and publication reviews and more discussion of good analysis practices are needed. Although myEpi is focused on the individual, it has important potential in the improvement of public health by acting on *one person at a time*. The application areas could be different (e.g., monitoring sleep, blood pressure, food and exercise, tracking cigarettes smoked) and the results will vary drastically by the application, tool, and individual. However, my hypothesis is that those individuals who use myEpi (i.e., monitor their health using intelligent tools and rigorous methods) would improve their well-being more than those who do not.

## Conflict of Interest Statement

The author declares that the research was conducted in the absence of any commercial or financial relationships that could be construed as a potential conflict of interest.
